# Gabapentin for the Management of Suspected Cannabis Withdrawal in a Pregnant Patient With a History of Cannabinoid Hyperemesis Syndrome: A Case Report

**DOI:** 10.1155/crog/3043344

**Published:** 2026-09-01

**Authors:** Anne-Marie Turcotte, Charlotte Lemieux-Bourque, Catherine McCarey, Katéri Lévesque, Geneviève Kerkerian, Audrey Ann Labrecque

**Affiliations:** ^1^ Department of Obstetrics and Gynecology, University of Montreal, Montreal, Quebec, Canada, umontreal.ca; ^2^ Department of Obstetrics and Gynecology, CHU Sainte-Justine, Montreal, Quebec, Canada, chusj.org; ^3^ Department of Obstetrics, Department of Women, Children and Adolescents, University Hospitals of Geneva, Geneva, Switzerland, hug-ge.ch; ^4^ Division of Social Medicine, Department of Medicine, Providence Health Care, Vancouver, British Columbia, Canada, providencehealthcare.org

**Keywords:** cannabinoid hyperemesis syndrome, cannabis withdrawal syndrome, case reports, gabapentin, pregnancy

## Abstract

Cannabinoid hyperemesis syndrome (CHS) is a rare but increasingly recognized condition among chronic cannabis users, characterized by cyclic nausea, vomiting, and relief with hot bathing. During pregnancy, CHS may mimic hyperemesis gravidarum (HG), complicating diagnosis and management. We report the case of a 24‐year‐old pregnant patient who presented at 20 weeks′ gestation with recurrent nausea, vomiting, and labile hypertension. Investigations ruled out preeclampsia and secondary causes of hypertension. She met DSM‐5 criteria for cannabis use disorder, had a prior hospitalization for CHS, and had recently reduced her cannabis use, raising concern for withdrawal as a contributing factor to her presentation. Gabapentin was initiated off‐label after an Addiction Medicine consultation, resulting in resolution of symptoms and normalization of blood pressure within 24 h. To our knowledge, this is the first reported use of gabapentin in a pregnant patient with a history of CHS and suspected cannabis withdrawal. This report highlights the importance of screening for cannabis use, recent reduction or cessation, and withdrawal symptoms in pregnant patients presenting with severe or refractory hyperemesis. In this case, short‐term off‐label gabapentin use led to rapid symptom resolution without apparent maternal or fetal complications, suggesting that it may be relatively safe in this population, although data remain limited.

## 1. Introduction

Cannabinoid Hyperemesis Syndrome (CHS) is a clinical entity characterized by recurrent episodes of nausea, vomiting, and abdominal pain in the context of chronic cannabis use. It is often refractory to standard antiemetic therapies such as diphenhydramine, ondansetron, or metoclopramide, and is classically associated with symptom relief through hot baths or showers [1]. In the Canadian context, where cannabis has been legalized since 2018, its use has significantly increased across the general population, reaching approximately 27% as of 2024 [2]. A notable and statistically significant increase has also been observed among women of reproductive age, with prevalence rising from 18% in 2018 to 23% in recent years [2]. These trends pose not only a public health concern, but also a growing diagnostic and management challenge in clinical obstetrics.

The overlap between symptoms of CHS and those of hyperemesis gravidarum (HG) can make diagnosis particularly challenging during pregnancy. Nausea and vomiting affect up to 80% of pregnant women, and HG is diagnosed in approximately 0.3%–3% of pregnancies [3]. In this context, CHS‐related symptoms are frequently misattributed to pregnancy itself, potentially delaying appropriate management. This diagnostic complexity may be further increased when recent cannabis reduction or cessation raises concern for overlapping withdrawal symptoms.

We present the case of a pregnant patient with refractory nausea, vomiting, and labile hypertension, whose symptoms resolved rapidly following off‐label initiation of gabapentin for suspected cannabis withdrawal in the context of a history of CHS. To our knowledge, this clinical scenario has not been previously reported in the literature.

## 2. Case Presentation

### 2.1. Patient History and Initial Presentation

A 24‐year‐old G1P0 woman was referred to our tertiary care center at 20 + 2 weeks′ gestation for a detailed fetal ultrasound following an elevated maternal serum alpha‐fetoprotein (*α*FP: 6 MoM). Aside from presumed endometriosis and untreated anxiety disorder, her medical history was unremarkable. She had no surgical history or known allergies. Her only regular medication was pyridoxine‐doxylamine (two tablets four times daily). Regarding lifestyle factors, she denied alcohol use but reported daily smoked cannabis consumption early in pregnancy (4–6 g/day), recently reduced to approximately 1 g/day. To compensate, she increased her tobacco use. She lived with her sister and boyfriend, both of whom smoked cannabis daily inside their apartment, contributing to passive exposure.

Before her scheduled ultrasound, the patient experienced a syncopal episode in the hospital′s bathroom. Upon further questioning, she described recurrent nausea, epigastric pain, and vomiting since early pregnancy, associated with occasional presyncopal episodes, intermittent headaches, and minimal weight gain. These symptoms were only minimally relieved by her antiemetic medication. On initial examination, she was afebrile with normal oxygen saturation. Blood pressure (BP) was 148/90 mmHg and heart rate (HR) was 95 bpm. Given these findings, she was admitted for further evaluation.

That night, her BP peaked at 180/87 mmHg with associated temporal headache and transient scotomas. Neurological examination was unremarkable. Two doses of labetalol 100 mg were administered, lowering BP to 110/70 mmHg and HR to 76 bpm.

### 2.2. Investigations

The patient was managed using our institutional HG protocol, consisting of bowel rest (NPO), multivitamin infusion (B1 and B6), as well as intravenous administration of diphenhydramine, ranitidine, and metoclopramide for symptom management. Initial preeclampsia workup was normal.

Given her complaint of episodic abdominal pain, an abdominal ultrasound was performed the next day and was unremarkable. A detailed fetal ultrasound showed no fetal or placental anomalies. However, during the appointment, the patient experienced another hypertensive spike (BP: 181/89 mmHg) with presyncope. She received a single dose of short‐acting nifedipine 5 mg and was started on nifedipine XL 30 mg once daily, plus labetalol 100 mg as needed.

Given her history of nausea, vomiting and headaches, a noncontrast head CT‐scan was performed to rule out intracranial pathology and came back normal. Due to persistent episodes of severe hypertension despite antihypertensive treatment, severe early‐onset preeclampsia remained a concern. She was therefore transferred to an intermediate care unit and started on intravenous magnesium sulfate infusion for seizure prophylaxis while diagnostic investigations were ongoing.

The obstetrics and gynecology internal medicine service was consulted for evaluation of secondary hypertension. All investigations (TSH, serum calcium, renal Doppler ultrasound, urinary catecholamines and metanephrines, and aldosterone–renin ratio) were unrevealing. Repeat preeclampsia workup remained normal, and secondary causes of hypertension were not identified. Because the patient had reduced but not discontinued cannabis use, the positive urine toxicology screen for cannabinoids (cut‐off: 50 mg/mL of 11‐nor‐9‐carboxy‐delta‐9‐THC) was expected but did not exclude withdrawal. The screen was negative for other substances.

Further history‐taking revealed important contextual details. The patient reported that nausea and vomiting worsened at work and improved in the morning. She reported relief by hot baths or showers. Importantly, she disclosed a prior hospitalization for CHS and hypertension before conception. Her history met DSM‐5 criteria for cannabis use disorder (CUD) [4], and recent cannabis reduction raised suspicion for a withdrawal syndrome. Finally, the patient reported that her household context made her feel considerable social pressure regarding her own consumption. Given this context, her overall precarious social situation, psychiatric, and social work were consulted.

### 2.3. Treatment and Management

Given the strong suspicion that cannabis withdrawal was contributing to the patient′s current symptoms, the maternal–fetal medicine team consulted with an addiction medicine specialist from another Canadian tertiary care center. Based on the clinical history, including the recent substantial reduction in cannabis use, the consultant recommended initiating gabapentin, an off‐label option occasionally used in her department to alleviate cannabis withdrawal symptoms. Although not officially approved for this indication, gabapentin has shown promise in mitigating withdrawal symptoms in nonpregnant populations [5] and is considered relatively safe in pregnancy, particularly when used short‐term [6].

Gabapentin was initiated on Day 2 of her hospitalization at a dose of 300 mg orally three times daily (TID). The patient tolerated the treatment well, without adverse effects. She was advised to avoid direct and passive cannabis exposure to prevent recurrence of symptoms. At discharge, a short tapering schedule was prescribed to discontinue the medication: Gabapentin 300 mg PO TID for 4 days, then 300 mg PO BID for 2 days, followed by 300 mg PO once daily for 2 days, then discontinued.

### 2.4. Outcome

The patient showed marked clinical improvement within 24 h of initiating gabapentin, with resolution of nausea, vomiting, and general malaise. Her BP also normalized, and she was able to resume oral intake. Given the normal preeclampsia workup, absence of end‐organ involvement, and sustained clinical stability, magnesium sulfate was discontinued and the diagnosis of preeclampsia was not retained. She was transferred back to the maternal–fetal medicine care unit and remained stable without further antihypertensive therapy on the day prior to discharge. She was discharged on hospital Day 4 in stable condition, with follow‐up arranged with her primary obstetrical team. Attempts to contact her treating physician for pregnancy outcome were unsuccessful.

## 3. Discussion

This case underscores the importance of recognizing CUD in pregnant patients, per DSM‐5 criteria, and highlights the value of addiction medicine consultation for withdrawal management. To our knowledge, this is the first report discussing gabapentin use for suspected cannabis withdrawal in a pregnant patient with a history of CHS. This medication, approved for treatment of neuropathic pain, seizures, and restless legs syndrome, is considered relatively safe in pregnancy when used short‐term, with no clear association with first‐trimester malformations [6]. In this case, short‐term gabapentin use was associated with rapid withdrawal‐symptom resolution without apparent maternal or fetal complications. This suggests a potential role for this agent in selected cases where withdrawal symptoms are suspected, but this observation should be interpreted cautiously, and further studies are needed to confirm safety and efficacy.

Compared with previously published cases of CHS in pregnancy, our patient shared several typical features, including heavy cannabis use, recurrent nausea and vomiting with limited response to conventional antiemetics, and symptom relief with hot bathing [1]. As in prior reports, these overlapping symptoms initially raised concern for HG, illustrating how CHS may be overlooked during pregnancy and contribute to diagnostic delays [7]. This supports the importance of comprehensive substance use screening when symptoms are atypical, severe, or refractory to standard treatment.

However, this case differs from prior reports in two important ways. First, the recent substantial reduction in cannabis use raised concern for overlapping cannabis withdrawal rather than CHS alone. Unlike prior pregnancy cases, which primarily focused on cannabis cessation and supportive antiemetic management [1], this case describes short‐term gabapentin use specifically targeting suspected withdrawal symptoms in pregnant patient with a history of CHS. This distinction is important because CHS typically improves with cannabis cessation, whereas withdrawal symptoms may emerge or worsen after abrupt reduction or cessation [8]. In our patient, conventional antiemetic therapy had provided limited relief, and re‐exposure to cannabis or cannabinoid agonists was not appropriate during pregnancy [9]. Gabapentin was therefore considered after an addiction medicine consultation as a short‐term off‐label option for withdrawal management. Although not formally approved for this indication, gabapentin has shown promise in alleviating cannabis withdrawal symptoms in nonpregnant populations [5] and is used by addiction medicine specialists for management of symptomatic cannabis withdrawal in patients with severe cannabis use disorder. Its mechanism of action in this context remains incompletely understood, but its potential benefit is thought to relate, at least in part, to modulation of the GABA/glutamate dysregulation that occurs during cannabis withdrawal. Gabapentin binds to the alpha‐2d subunit of voltage‐gated calcium channels, thereby reducing presynaptic calcium influx and decreasing excitatory neurotransmitter release, particularly glutamate [10] while also indirectly modulating GABAergic signaling [11]. Cannabis withdrawal is believed to involve disruption of GABA/glutamate homeostasis, partly related to CB1 receptor desensitization and downregulation following chronic cannabis exposure [12]. Therefore, gabapentin mechanism may help restore balance within dysregulated brain stress systems and alleviate withdrawal‐related symptoms [5]. In addition, a study by Lile et al. [13] reported that gabapentin may produce interoceptive effects similar to cannabinoids, which could further contribute to its potential benefit in a withdrawal setting.

Secondly, the presentation was complicated with labile severe‐range hypertension, which raised concern for early‐onset preeclampsia and prompted magnesium sulfate administration while investigations were ongoing. Although hypertension is not considered a cardinal feature of cannabis withdrawal, transient BP elevations have been described after abrupt cessation or reduction of heavy cannabis use [14]. THC exposure affects cardiovascular regulation through CB1 receptor‐mediated vasodilatation and modulation of peripheral sympathetic outflow [15]. As mentioned previously, CB1 receptors are downregulated following chronic cannabis use [12]. This may contribute to autonomic instability when cannabis is suddenly discontinued and result in a rebound increase in cardiovascular parameters, including BP. These effects present a diagnostic challenge during pregnancy, where such findings may mimic preeclampsia and lead to unnecessary interventions. In our patient, the subsequent BP normalization, together with the absence of evidence of preeclampsia, further supports the hypothesis of cannabis withdrawal‐induced transient hypertension, although causality cannot be established from a single case.

**Nomenclature**
CHS: cannabinoid hyperemesis syndrome
CUD: cannabis use disorder
HG: hyperemesis gravidarum

## Funding

No funding was received for this manuscript.

## Consent

The patient, whose story is told in this report, gave written consent for its publication.

## Conflicts of Interest

The authors declare no conflicts of interest.

## Data Availability

The data that support the findings of this study are available on request from the corresponding author. The data are not publicly available due to privacy or ethical restrictions.

## References

[1] Hanley S. , Imcha M. , and Mohamad M. M. , Cannabinoid Hyperemesis Syndrome in Pregnancy: A Case Series and Review, Obstetric Medicine. (2025) 18, no. 4, 264–271, 10.1177/1753495X241307415, 39759763. 39759763 PMC11694266

[2] Canada S , Canadian Cannabis Survey (CCS) 2024, https://www.canada.ca/en/health-canada/services/drugs-medication/cannabis/research-data/canadian-cannabis-survey-2024-summary.html.

[3] Niebyl J. R. , Nausea and Vomiting in Pregnancy, New England Journal of Medicine. (2010) 363, no. 16, 1544–1550, 10.1056/NEJMcp1003896. 20942670

[4] Patel J M. R. , Cannabis Use Disorder, StatPearls [Internet], 2025, StatPearls Publishing, https://www.ncbi.nlm.nih.gov/books/NBK538131/.

[5] Mason B. J. , Crean R. , Goodell V. , Light J. M. , Quello S. , Shadan F. , Buffkins K. , Kyle M. , Adusumalli M. , Begovic A. , and Rao S. , A Proof-of-Concept Randomized Controlled Study of Gabapentin: Effects on Cannabis Use, Withdrawal and Executive Function Deficits in Cannabis-Dependent Adults, Neuropsychopharmacology. (2012) 37, no. 7, 1689–1698, 10.1038/npp.2012.14, 22373942. 22373942 PMC3358737

[6] Patorno E. , Hernandez-Diaz S. , Huybrechts K. F. , Desai R. J. , Cohen J. M. , Mogun H. , and Bateman B. T. , Gabapentin in Pregnancy and the Risk of Adverse Neonatal and Maternal Outcomes: A Population-Based Cohort Study Nested in the US Medicaid Analytic Extract Dataset, PLoS Medicine. (2020) 17, no. 9, e1003322, 10.1371/journal.pmed.1003322, 32870921. 32870921 PMC7462308

[7] Kirby J. and Naren T. , Cannabinoid Hyperemesis Syndrome in Pregnancy: Challenges and Opportunities, Australian and New Zealand Journal of Obstetrics and Gynaecology. (2023) 63, no. 6, 746–752, 10.1111/ajo.13714, 37259610. 37259610

[8] Rubio-Tapia A. , McCallum R. , and Camilleri M. , AGA Clinical Practice Update on Diagnosis and Management of Cannabinoid Hyperemesis Syndrome: Commentary, Gastroenterology. (2024) 166, no. 5, 930–934, 10.1053/j.gastro.2024.01.040, 38456869. 38456869

[9] Valent A. M. , Russo M. L. , and Lawson S. M. , ACOG Clinical Consensus No. 10: Cannabis Use During Pregnancy and Lactation, Obstetrics & Gynecology. (2025) 146, no. 4, 600–611, 10.1097/AOG.0000000000006053. 40966737

[10] Quintero J. E. , Dooley D. J. , Pomerleau F. , Huettl P. , and Gerhardt G. A. , Amperometric Measurement of Glutamate Release Modulation by Gabapentin and Pregabalin in Rat Neocortical Slices: Role of Voltage-Sensitive Ca2+ *α*2*δ*-1 Subunit, Journal of Pharmacology and Experimental Therapeutics. (2011) 338, no. 1, 240–245, 10.1124/jpet.110.178384, 21464332. 21464332 PMC3126634

[11] Petroff O. A. , Hyder F. , Rothman D. L. , and Mattson R. H. , Effects of Gabapentin on Brain GABA, Homocarnosine, and Pyrrolidinone in Epilepsy Patients, Epilepsia. (2000) 41, no. 6, 675–680, 10.1111/j.1528-1157.2000.tb00227.x, 10840398. 10840398

[12] Zuo C. S. , Davis K. A. , Kuppe M. K. , Dahlgren M. K. , Gruber S. , Fitzmaurice G. M. , and Lukas S. E. , Elevated Striatal Glutamate + Glutamine in Recreational Cannabis Users During Abstinence, Journal of Psychiatric Research. (2022) 146, 192–200, 10.1016/j.jpsychires.2021.12.041, 34999370. 34999370 PMC8803139

[13] Lile J. A. , Wesley M. J. , Kelly T. H. , and Hays L. R. , Separate and Combined Effects of Gabapentin and [INCREMENT]9-Tetrahydrocannabinol in Humans Discriminating [INCREMENT]9-Tetrahydrocannabinol, Behavioural Pharmacology. (2016) 27, no. 2 and 3, 215–224, 10.1097/FBP.0000000000000187. 26313650 PMC4769128

[14] Vandrey R. , Umbricht A. , and Strain E. C. , Increased Blood Pressure After Abrupt Cessation of Daily Cannabis Use, Journal of Addiction Medicine. (2011) 5, no. 1, 16–20, 10.1097/ADM.0b013e3181d2b309, 21359104. 21359104 PMC3045206

[15] Cunha P. , Romão A. M. , Mascarenhas-Melo F. , Teixeira H. M. , and Reis F. , Endocannabinoid System in Cardiovascular Disorders - New Pharmacotherapeutic Opportunities, Journal of Pharmacy and Bioallied Sciences. (2011) 3, no. 3, 350–360, 10.4103/0975-7406.84435, 21966155. 21966155 PMC3178941

